# The Localization Accuracy of Electrically Evoked Dental Sensations

**DOI:** 10.1002/cre2.70012

**Published:** 2025-02-27

**Authors:** Nuha Ab Ashaibi, Andrew Graham Mason, Mark Prichard Hector, Pauline Maillou

**Affiliations:** ^1^ School of Dentistry University of Dundee Dundee UK

**Keywords:** dental, localization, pain

## Abstract

**Objectives:**

Dental pain of odontogenic origin can be difficult to accurately locate to a specific tooth. This study aimed to determine how effectively volunteers could locate electrical stimuli applied to teeth in both the anterior and posterior regions of the upper arch.

**Materials and Methods:**

This study was approved by the University of Dundee Research Ethics Committee (No. UREC15068). Experiments were performed on 19 healthy consenting volunteers (2 male and 17 female). Customized tooth electrodes were temporarily attached to five teeth, allowing single 1‐ms electrical stimuli at two intensities (pain threshold [T] and suprathreshold [1.25T]) to be delivered randomly to any one of three teeth (three test teeth and two controls). Volunteers were asked to indicate the location of the stimulus by pointing with their fingers. This was repeated 18 times (nine times with two different stimulus intensities) in a random order using a counterbalancing scheme. Three sessions were required to include all test teeth.

**Results:**

The overall accuracy of localization anteriorly was 67% at intensity T and 66% at 1.25T. For the right and left posterior teeth, the accuracy was 55% and 47% at T and 44% and 42% at 1.25T, respectively. No statistically significant differences were detected at T (*p* = 0.35, Bonferroni‐corrected Mann–Whitney *U* test, *α* = 0.01) and at 1.25T (*p* = 0.28, Bonferroni‐corrected Mann–Whitney *U* test, *α* = 0.01).

**Conclusions:**

Odontogenic pain was poorly localized even under highly controlled experimental conditions. Pain in posterior teeth may be more difficult to correctly locate compared to anterior teeth, particularly at higher intensities.

## 1 Introduction

The localization accuracy of odontogenic pain is crucial for oral diagnosis (Thayer 2015). Patients may present with an inability to precisely localize the affected tooth; furthermore, a study by Mardani, Eghbal, and Baharvand (2008) showed that more than half of dental pain presentations originating from the dental pulp were referred to other oral structures. Although dentine and pulpal tissues are highly innervated, it is challenging to locate a painful stimulus to a specific tooth when these structures are stimulated. This difficulty is supported by anatomical and physiological evidence whereby this clinical problem has its foundation in the branching of primary afferent nerves supplying tooth pulps and the divergence of primary afferent nerves before the first synapse in the brainstem nuclei (Lisney and Matthews 1978; Foster and Robinson 1993; Cadden and Orchardson 2001). Conversely, localization is easier when inflammation involves the periapical region, as this activates receptors of the periodontal ligament, which can present as the tooth being tender to percussion (Bender 2000).

Experimentally, tooth pain can be evoked by electrical stimulation of teeth (Cadden, Mason, and Van Der Glas 2013). The issue of poor localization of dental pain in humans has been addressed in previous studies using monopolar electrical stimulation (Friend and Glenwright 1968; Van Hassel and Harrington 1969; Brodison 1995; Bishop 2001). With monopolar stimulation, the electrical current that is delivered to the crown of the tooth passes through the pulp but may also pass to the periodontal tissue unless a very careful and specific protocol is followed (Matthews et al. 1974; Jafarzadeh and Abbott 2010). In support of this, it has been shown that monopolar stimuli applied to pulpless, endodontically treated teeth are capable of eliciting a sensation due to stimulus spread beyond the tooth (Matthews, Baxter, and Watts 1976). Bipolar stimulation is preferable and more reliable than monopolar stimulation for two main reasons: (a) it has been shown that, even at high intensities, bipolar stimulation do not elicit any sensory or reflex responses from non‐vital teeth compared to vital teeth (Matthews, Baxter, and Watts 1976; Matthews and Searle 1976; Cadden, Mason, and Van Der Glas 2013); and (b) customized bipolar electrodes have been shown to maintain stable electrical properties throughout the experiment, thereby delivering a consistent stimulus intensity to the participant (Cadden, Mason, and Van Der Glas 2013).

The primary aim of this study was to investigate the accuracy of localization of experimentally evoked pulpal pain in the anterior and bilateral posterior maxillary teeth using customized bipolar electrodes. A secondary aim was to compare this pain localization ability between dentally knowledgeable individuals (dentists and students) and dentally unknowledgeable/naive volunteers.

## 2 Materials and Methods

### 2.1 Volunteer Participants

The study was approved by the University of Dundee Research Ethics Committee (No. UREC15086). A convenience sample of 19 healthy, dentate volunteers took part in this study: 10 with dental knowledge and 9 without dental knowledge. There were 17 females and 2 males, with ages ranging from 19 to 37 years (median age 27 years). The following exclusion criteria were used: those who had taken any medication and/or analgesic medication within 4 h of the experiment commencing, those who were allergic to any of the materials used in the experiment, those with an embedded electrical device, those who had undergone root canal treatment or major restorations in the teeth to be stimulated, and pregnant women. Written informed consent was obtained from each volunteer participant before the study commenced.

### 2.2 Tooth Electrodes

**Figure 1:**
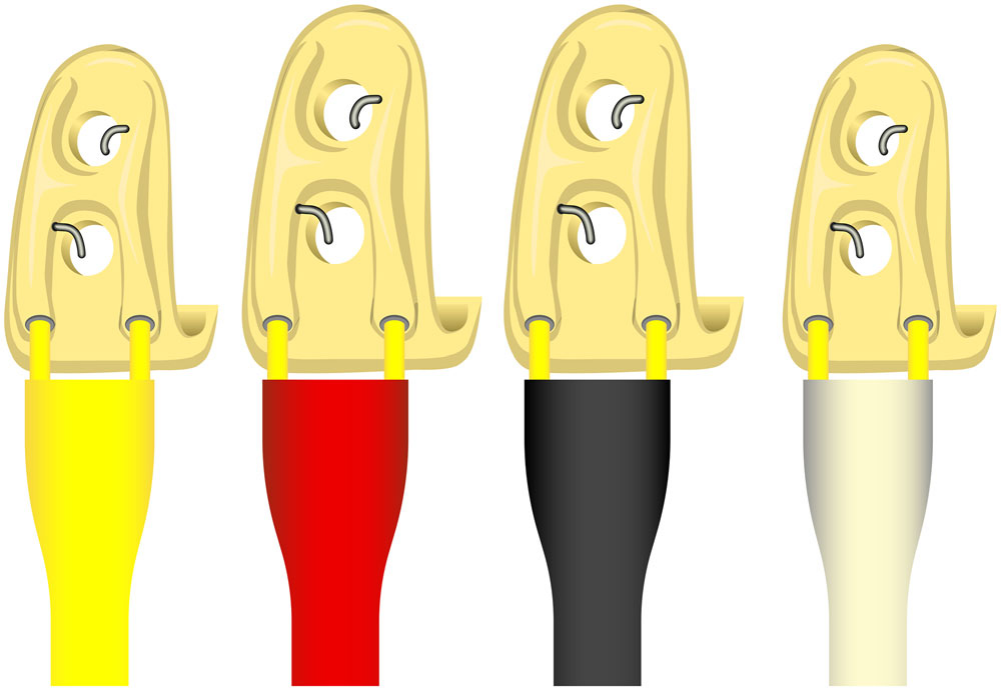
This figure shows the different colors of heat shrinkage for each electrode, from left to right: 13, 11, 21, and 22.

Customized bipolar tooth electrodes were used to deliver the electric current to the teeth. These electrodes were designed at the School of Dentistry, University of Dundee, with their construction and validation described in detail by Cadden, Mason, and Van Der Glas (2013). In summary, a maxillary impression was recorded using a condensation‐cured dental silicone impression material (Zetaplus, Zhermack, Italy). This impression was cast using dental stone, and the electrodes were constructed on the five teeth involved in the experiment. The electrodes were fabricated on the stone model. A thin layer (1 mm) of light‐cured submicron hybrid composite resin material (Spectrum TPH3, Dentsply, Weybridge, UK) was applied to the labial/buccal surface of each tooth, and two holes were created, each 1.5 mm in diameter and 2 mm apart. A silver wire (AG15W, 0.37 mm, Clark Electromedical Instruments, UK) was inserted into each of these holes. Each tooth electrode had a specific color‐coded heat shrink insulation (Figure 1).

The electrodes were temporarily attached to the labial/buccal surface of selected teeth using calcium hydroxide dental lining material (Dycal, Dentsply, Weybridge, UK), ensuring that the holes were clear of the lining material. Electroconducting paste (Sensodyne Pronamel toothpaste, GSK, UK) was injected into the holes, giving an electrode contact area of 1.77 mm² per electrode. Whenever possible, the anode electrode was placed incisally/occlusally, and the cathode was placed cervically. Good moisture control was achieved using low‐volume suction (CA‐MI, Pilastro, Italy). Lip and cheek retractors (OptiView, Kerr Dental, Uxbridge, UK) or cotton wool rolls (Roeko Parotisroll, Coltene/Whaledent Ltd., Burgess Hill, UK) were used to further retract the cheek and lip away from the tooth, thereby improving accessibility.

### 2.3 Experimental Setup

**Figure 2:**
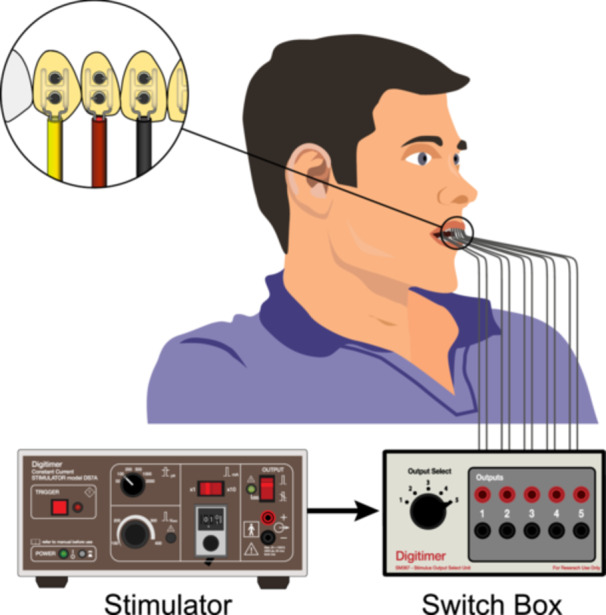
Experimental setup: Five customized tooth electrodes are attached to the teeth—three test teeth (green) and two control teeth (blue). They are connected to the switch box, which is connected to the DS7 stimulator.

The volunteers were seated in an upright position in the dental chair and five customized tooth electrodes were temporarily attached to the labial/buccal surfaces: three teeth were test teeth (stimuli were delivered to these teeth), and the other two were controls (stimuli were not delivered to these teeth). A constant current stimulator (Digitimer DS7, Digitimer Ltd., Welwyn Garden City, UK) was used to deliver electrical stimuli to the teeth through the bipolar tooth electrodes (Figure 2). A manual switcher unit (D‐SP367, Digitimer Ltd., Welwyn Garden City, UK) was connected between each customized tooth electrode and the DS7 stimulator. This unit had five outputs to select which tooth would be stimulated.

### 2.4 Experimental Protocol

**Figure 3:**
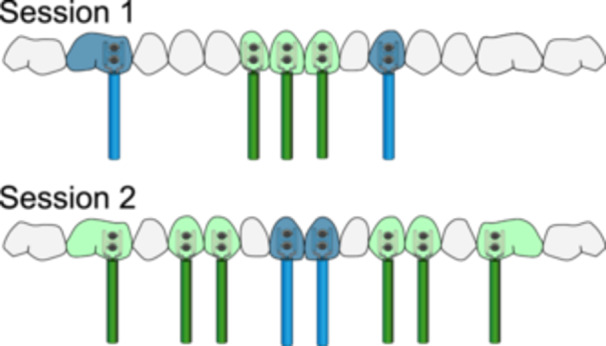
Experimental teeth: Green teeth are test teeth and blue teeth are control teeth. Session 1: Incisor test teeth and control teeth. Session 2: Teeth posterior to the incisor teeth are test teeth (L/R) and anterior teeth are control teeth.

The experiment utilized maxillary teeth due to ease of isolation, size of the crowns for electrode fabrication and to be comparable to previous studies (Friend and Glenwright 1968; Brodison 1995). The teeth chosen gave a range of morphologies of crown shape and root configurations. Three experimental sessions were required to evaluate all test teeth: one session for maxillary incisor teeth and the other two sessions for the left and right maxillary teeth posterior to the incisors (canines, first premolars, and first molars). Each session lasted between 30 and 60 min. For the incisor teeth experimental session, the test teeth were 12, 11, and 21, whereas the control teeth were 16 and 23. For the more posterior teeth experimental session, the test teeth were either 13, 14, and 16 or 23, 24, and 26. The control teeth were 11 and 21 (Figure 3).

### 2.5 Determination of Sensory and Pain Threshold

After attaching all the electrodes to the teeth, one test stimulus was delivered to ensure that the volunteer became familiar with the sensation. The sensory and pain thresholds were determined. The sensory threshold is the “pre‐pain sensation” that was defined by Shimizu (1964) as the “weakest sensation elicited by electrical stimulation of human teeth.” When the stimulus intensity was increased, this non‐painful sensation becomes sharp, and the lowest intensity at which the subject perceived a sharp sensation was called the pain threshold (T). These thresholds were determined using the “Staircase Limit” protocol (Jacobs et al. 2002; Cadden, Mason, and Van Der Glas 2013).

The pain threshold was multiplied by 1.25 to achieve suprapain intensity (1.25T), which was used along with the pain threshold (T) for the determination of accuracy of location in the subsequent part of the experiment.

### 2.6 Recording the Accuracy of Localization

Electrical stimuli, at pain threshold (T) and suprapain threshold (1.25T), were delivered randomly to each of the three test teeth. The volunteer was not told which tooth was being stimulated, and, furthermore, was expecting a stimulus to be delivered to any of the five teeth that had electrodes attached (test and control teeth). The volunteer was asked to indicate the location of the sensation by pointing to the tooth that they perceived was being stimulated. The two intensities (T and 1.25T) were delivered in a random order according to a counterbalancing scheme (Zeelenberg and Pecher 2015). Therefore, each tooth was stimulated six times: three times at T and three times at 1.25T.

The percentage success of localization was calculated for every volunteer at intensities T and 1.25T in each session, including anterior, left, and right posterior teeth, as follows:

$$\frac{\mathrm{number}\;\mathrm{of}\;\mathrm{correctly}\;\mathrm{identified}\;\mathrm{stimuli}}{\mathrm{total}\;\mathrm{number}\;\mathrm{of}\;\mathrm{stimuli}}\times 100$$

### 2.7 Statistical Analysis

Data were analyzed using nonparametric statistical methods (SPSS V22 Software, IBM Corporation, New York, USA). The median percentage success rates for each tooth at both intensities (T and 1.25T) were compared using Friedman's test with Bonferroni correction of 3. To compare the overall percentage success rates between anterior and bilateral posterior teeth for each group (dentally knowledgeable and dentally unknowledgeable volunteers), Friedman's test of differences among repeated measures was carried out with a Bonferroni correction of 2.

The overall accuracy of localization at both intensities T and 1.25T and the comparison between dentally knowledgeable and dentally unknowledgeable volunteers were carried out using the Mann–Whitney *U* test conducted with Bonferroni correction of 3 to allow multi‐comparison nature of these analyses. The *p* values less than 0.05 were considered significant, with the *α*‐value Bonferroni‐corrected for each test.

Data are presented as the outcome of the statistical test (*p* value) and the Bonferroni‐corrected *α*‐value as the reference for statistical significance.

## 3 Results

### 3.1 The Accuracy of Location of Experimental Pain for Each Stimulated Tooth

**Figure 4:**
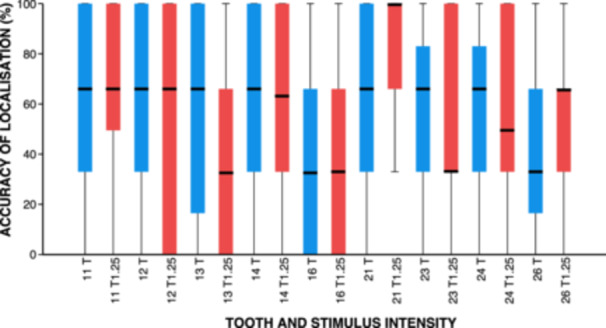
A box and whisker plot illustrating the percentage success rate for the pooled data for each tooth.

The median success percentage for each tooth for all volunteers was calculated, which is presented in Table 1 and illustrated in Figure 4. There were no statistically significant differences of success rate between teeth at intensity T (*p* = 0.34, Bonferroni‐corrected Friedman's two‐way ANOVA, *α* = 0.01) and at 1.25T (*p* = 0.60, Bonferroni‐corrected Friedman's two‐way ANOVA, *α* = 0.01), and between both intensities (*p* = 0.41, Bonferroni‐corrected Friedman's two‐way ANOVA, *α* = 0.01).

**Table 1:** The median percentage success rate for each tooth.

Tooth	T (%)	1.25T (%)
11	66	66
12	66	66
13	66	33
14	66	66
16	33	33
21	66	100
23	66	33
24	66	50
26	33	66

### 3.2 The Accuracy of Location of Experimental Pain for Incisor Teeth Compared to Teeth Posterior to the Incisors

For non‐dental volunteers, the success percentages were higher for the incisor teeth compared to the other teeth, with rates of 72% and 33%, respectively (Table 2). However, the difference between the incisor teeth compared to the other teeth failed to reach statistical significance at T (*p* = 0.03, Bonferroni‐corrected Friedman's two‐way ANOVA, *α* = 0.025) and at 1.25T (*p* = 0.13, Bonferroni‐corrected Friedman's two‐way ANOVA, *α* = 0.025).

**Table 2:** The overall median percentage success for the localization comparing incisor teeth and teeth posterior to the incisors in dental and non‐dental volunteer subjects.

	Incisor teeth		Right side posterior to incisor teeth		Left side posterior to incisor teeth	
	T (%)	1.25T (%)	T (%)	1.25T (%)	T (%)	1.25T (%)
% success for non‐dental volunteers	72	72	40	33	40	40
% success for dental volunteers	66	66	73	67	67	66
Overall median % success	67	66	55	44	47	42

In dental volunteers, the median success percentage ranged between 66% for the incisor teeth and 73% for the other teeth (Table 2). The difference between the two areas failed to reach statistical significance at both intensities: T (*p* = 0.54, Bonferroni‐corrected Friedman's two‐way ANOVA, *α* = 0.025) and 1.25T (*p* = 0.88, Bonferroni‐corrected Friedman's two‐way ANOVA, *α* = 0.025).

**Figure 5:**
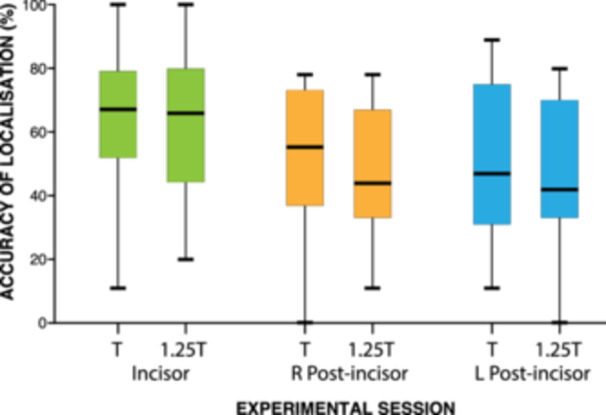
Box and whisker plot representing the median success rate for incisor teeth and teeth posterior to the incisor teeth at both intensities for all volunteers. Green: anterior teeth; orange: right post‐incisor teeth; blue: left post‐incisor teeth. Anterior = incisor teeth at T and 1.25T; L post = left post‐incisor teeth at T and 1.25T; R posterior = right post‐incisor teeth at T and 1.25T.

The data were pooled together and are illustrated in Table 2 and Figure 5. Subjectively, the accuracy of localization anteriorly was greater than posteriorly and ranged between 67% and 42%.

**Figure 6:**
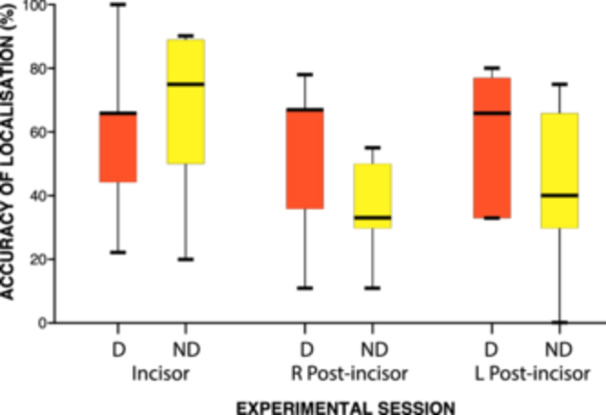
Box and whisker plot representing the median success rate for incisor teeth and teeth posterior to incisor teeth at the higher intensity (1.25T) for dental (D) and non‐dental (ND) volunteers.

The success rate of localization appears to be higher at intensity T (anterior 67%, right posterior 55%, left posterior 47%) than at intensity 1.25T (anterior 66%, right posterior 44%, left posterior 42%). The difference between the two intensities failed to show any statistically significant differences anteriorly and bilaterally posteriorly between dentally knowledgeable and dentally unknowledgeable volunteers at T (*p *= 0.35, Bonferroni‐corrected Mann–Whitney *U* test, *α* = 0.01) and at 1.25T (*p *= 0.28, Bonferroni‐corrected Mann–Whitney *U* test, *α* = 0.01). The data for the higher stimulus intensity (1.25T) comparing dental and non‐dental volunteers are presented in Figure 6.

## 4 Discussion

The results from this investigation showed that dental pain is poorly located. This finding is in agreement with Friend and Glenwright (1968), Brodison (1995), and Bishop (2001). However, the percentage success rates vary between the aforementioned studies, and this could be explained due to the different methodologies being employed. For example, the previous studies used monopolar stimulation, whereas this current study utilized bipolar stimulation. Furthermore, the number of teeth stimulated and the number of stimuli delivered per tooth varied between all studies. Friend and Glenwright (1968) showed that the accuracy of localization in maxillary teeth was 38.2%, Brodison (1995) found that the accuracy of localization in maxillary anterior teeth was 64% ± 8.87%, and Bishop (2001) reported that an overall success rate including maxillary and mandibular teeth was 36.8%.

In this study, both dentally knowledgeable and dentally unknowledgeable volunteers were included mainly to determine whether having dental background knowledge would make it easier to correctly locate evoked dental pain. Dentally unknowledgeable volunteers performed in a similar way to dentally knowledgeable volunteers in correctly identifying the stimulus location, and there were no statistically significant differences between them. This was also in agreement with Bishop (2001).

In terms of success percentage for each stimulated tooth, it was noticeable that the first molar tooth showed a trend to have the lowest subjective success rate compared to the other teeth. It was shown by Friend and Glenwright (1968) and Brodison (1995) that the success rate for localization of the central incisor tooth was higher than the other teeth. They found that the success percentages of localization for the upper central and lateral incisors were 62.5% and 42.5%, respectively. In this study, the percentage obtained was 66% for tooth 11 and 66% for tooth 12. For the other teeth involved in this study—canine, first premolar, and first molar (Table 1)—our results were subjectively higher compared to the reported figures from Friend and Glenwright (1968) who found 35.8%, 25.8%, and 35.0%, respectively. These differences could be related to different methodologies used as in this study bipolar stimulation was used, whereas Friend and Glenwright used monopolar stimulation.

Poor localization of pain may be in part due to anatomical factors, for example, the branching of peripheral nerves to supply more than one tooth pulp (Orchardson and Cadden 2001). It is also possible that the poor localization accuracy may be due to “radiated pain” or “referred pain.” This may explain why dental pain was difficult to locate Cadden and Orchardson (2001). Moreover, it is worth noting that the percentages of successful localization of the stimuli tended to decrease with the higher intensities applied (1.25T) (Table 2). This was also demonstrated by Brodison (1995). At higher intensities, activation generated in the primary afferent neurons is more capable of exciting not only their own second‐order neurones but also more peripheral or adjacent second‐order neurones due to divergence and convergence phenomena, and consequently may be difficult to locate (Cadden and Orchardson 2001).

## 5 Conclusion

1. This study confirmed that the ability to accurately locate dental pain is poor.
2. Dental knowledge did not help to improve the accuracy of localization.

Further research has been ongoing to explore the physiological basis of the localization of dental pain and how to improve this ability, ultimately assisting the patient with timely diagnosis and appropriate management of their dental pain.

## Author Contributions

The study was conceived by Pauline Maillou, Nuha Ab Ashaibi, Andrew Graham Mason, and Mark Prichard Hector. Data collection and analysis were carried out by Nuha Ab Ashaibi. The manuscript was prepared by Nuha Ab Ashaibi, Pauline Maillou, and Andrew Graham Mason. Manuscript illustrations were created by Andrew Graham Mason. All authors were involved in review, editing, and final approval of the manuscript.

## Ethics Statement

The study was approved by the University of Dundee Research Ethics Committee (Reference Number: UREC15086).

## Conflicts of Interest

The authors declare no conflicts of interest.

## Data Availability

The data that support the findings of this study are available from the corresponding author upon reasonable request.
